# Jie-Yu Pill, A Proprietary Herbal Medicine, Ameliorates Mood Disorder-Like Behavior and Cognitive Impairment in Estrogen-Deprived Mice Exposed to Chronic Unpredictable Mild Stress: Implication for a Potential Therapy of Menopause Syndrome

**DOI:** 10.3389/fpsyt.2020.579995

**Published:** 2020-10-29

**Authors:** Xi-Dan Zhou, Xin-Jing Yang, Yu Zheng, Zong-Shi Qin, Wei Sha, Gang Chen, Zhang-Jin Zhang

**Affiliations:** ^1^Li Ka Shing (LKS) Faculty of Medicine, School of Chinese Medicine, The University of Hong Kong, Hong Kong, China; ^2^Department of Research and Development, Henan Taifeng Biological Technology Corporation Limited, Kaifeng, China; ^3^Interdisciplinary Institute for Personalized Medicine in Brain Disorders, Jinan University, Guangzhou, China; ^4^Department of Chinese Medicine, The University of Hong Kong-Shenzhen Hospital, Shenzhen, China

**Keywords:** Jie-Yu Pill, herbal medicine, menopause syndrome, psychiatric disorders, estrogen deprivation, ovariectomized mice

## Abstract

Jie-Yu Pill (JYP) is a proprietary herbal medicine initially developed to treat menstrual mood disorders. This study sought to determine whether JYP could alleviate menopausal psychiatric symptoms in ovariectomized (OVX) mice, an animal model of estrogen deprivation, exposed to chronic unpredictable mild stress (CUMS) and the underlying mechanisms in comparison with estrogen therapy. The OVX+CUMS mice were treated with 0.3 mg/kg estradiol (E_2_), 2.5 g/kg or 5 g/kg JYP for 36 days, and tested in multiple behavioral paradigms. Serum, uterus, and brain tissues were collected for the measurement of hypothalamus-pituitary-ovarian axis (HPO) and hypothalamus-pituitary-adrenal (HPA) axis hormones, γ-aminobutyric acid (GABA), glutamate, neurotrophins, and estrogen receptors. JYP and E_2_ had comparable efficacy in reducing anxiety- and depression-like behavior and cognitive impairment of the OVX+CUMS mice. E_2_ strikingly increased ratio of uterus to body weight of the OVX+CUMS mice, but JYP did not. Both agents suppressed HPO-axis upstream hormones, inhibited HPA-axis hyperactivity by reinstating hypothalamic GABA, restored hippocampal and prefrontal glutamate contents and its receptor expression in the OVX+CUMS mice. While JYP and E_2_ protected against decreases in hippocampal and prefrontal neurotrophins and estrogen receptors of the OVX+CUMS mice, unlike E_2_, JYP had no significant effects on these biomarkers in the uterus. These results suggest that JYP has comparable efficacy in ameliorating mood disorder-like behavior and cognitive impairment induced by a combination of estrogen deprivation and chronic stress in association with certain differential uterus-brain mechanisms compared to estrogen therapy. JYP may be a potential therapy for menopause-associated psychiatric disorders.

## Introduction

Menopause is an important and physiological process in a woman's life, manifesting as the permanent cessation of menstruation due to the failure of ovarian follicular activity (1). Multiple psychiatric symptoms, including anxiety, depression, and cognitive decline, are frequent complaints of menopausal women and have become a significant obstacle to the quality of their life (1, 2). Although estrogen therapy is the mainstay of the management of menopausal syndrome, its efficacy in improving psychiatric symptoms is limited and even controversial; long-term estrogen therapy even increases the risk of breast and ovarian cancer, stroke, and cardiovascular disease (3). Therefore, the development of novel treatment strategies for menopausal women is greatly desired.

Jie-Yu Pill (JYP), consisting of 10 herbal materials (Table 1), is a proprietary agent which was initially developed from classic Chinese medicine formulae to treat menstrual cycle related mood symptoms (4, 5). Clinical studies have shown the effectiveness of JYP in the treatment of depressive and anxiety disorders, including climacteric depression, postpartum depression, generalized anxiety disorder, and psychosomatic disorders (4, 6). A series of previous studies have confirmed anxiolytic and antidepressant effects of JYP in normal rodents and its modulatory effects on the hypothalamic-pituitary-adrenal (HPA) axis and monoamine neurotransmitters in the prefrontal cortex, hippocampus, and hypothalamus, the three brain regions that play key roles in the development of menopausal psychiatric disorders (7–12).

**Table 1:** Individual Medicinal materials of the JYP formula^a^.

**Material name (in Chinese)**	**%**	**Major bioactive constituents**
Paeoniae Radix Alba (Bai-Shao)	16.395	Paeoniflorin
Bupleuri Radix (Chai-Hu)	12.298	Saikosaponins A
Angelicae Sinensis Radix (Dang-Gui)	8.195	Ferulic Acid
Curcumae Radix (Yu-Jin)	8.195	Essential oils, curcuminoids
Poria (Fu-Ling)	9.835	Polysaccharides
Lily bulb (Bai-He)	9.835	Polysaccharides
Silktree Albizia Bark (He-Huan-Pi)	9.835	Albitocin
Wheat (Xiao-Mai)	12.298	
Licorice (Gao-Cao)	4.919	Liquiritin and glycyrrhizic acid
Chinese Date (Da-Zao)	8.195	Ziziphussaponins, jujuboside B

a *The detailed information can be seen at: https://patentimages.storage.googleapis.com/68/b2/47/ed1207e1332697/CN1404864A.pdf*.

On the other hand, one herbal medicine formula derived from JYP's two herbal materials, paeoniae radix and glycyrrhizae radix, could normalize dopamine hyperactivity induced female sex hormone dysfunction (13). Several constituents contained in JYP, such as paeoniflorin (14), saikosaponin A (15), and polysaccharides of lily bulb (*Lilium lancifolium* Thunb.) (16), could reduce estrogen deprivation induced anxiety, depression, and cognitive impairment in animal models. Total polysaccharides of lily bulb and estrogen therapy exerted differential modulatory effects on different neurotrophins and estrogen receptor subtypes in the uterus and brain regions (16, 17). In addition, brain γ-aminobutyric acid (GABA) and glutamatergic neuronal system are directly involved in the pathophysiology of psychiatric disorders occurred during menopausal transition (18–20). Brain neurotrophins modulate estrogen-associated learning, memory, and the underlying neuroplasticity (21, 22).

We therefore hypothesized that JYP could produce comparable efficacy in alleviating menopause-related psychiatric disorders compared to estrogen therapy in association with differential mechanisms at peripheral reproductive organs and the brain. Ovariectomized (OVX) animals are often used as a model of estrogen deprivation as it is well-validated to represent most estrogen deprivation-associated clinical features in the adult human (23). We have modified this model by exposing the OVX mice to chronic unpredictable mild stress (CUMS) to facilitate the development of mood disorder-like behavior and cognitive impairment (16, 17). In this study, the psychotropic effects of JYP were evaluated in behavioral paradigms of anxiety, depression, and cognition in OVX mice exposed to CUMS. We also examined the effects of JYP on serum hormones of the hypothalamus-pituitary-ovary (HPO) and HPA axes, the expression of GABA, glutamate, neurotrophins, estrogen receptor α (ERα) and β (ERβ) in the prefrontal cortex, hippocampus, and hypothalamus.

## Materials and Methods

### Animals and Experimental Time Course

All experimental procedures were approved by the Committee on the Use of Live Animals in Teaching and Research of the University of Hong Kong (CULATR 3812-15). A total of 50 C57BL/6N female mice weighing 18–22 g at 8 weeks of age were purchased from Charles River Laboratory (Wilmington, MA, USA). Mice were housed in 3–4 per cage at a constant temperature (23 ± 2°C) and maintained on a 12 h/12 h light/dark cycle (lights on 7:00–19:00) with *ad libitum* access to food and water.

**Figure 1:**
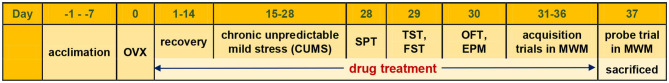
Experimental time course. OVX, ovariectomized; SPT, sucrose preference test; TST, tail suspension test; FST, forced-swimming test; OFT, open-field test; EPM, elevated plus maze; MWM, Morris water maze.

The experimental time course is illustrated in Figure 1. After 1 week of acclimatization, animals received OVX or sham surgery and were allowed for 2 weeks of recovery. Our preliminary study found that OVX itself could not consistently evoke remarkable psychiatric disorder-like behavior. Chronic unpredictable mild stress (CUMS) procedure was then added to accelerate the development of mood disorder-like behavior and cognitive impairment. Multiple behavioral tests were carried out at 4 weeks post-OVX. Treatment started at 1 day after OVX and throughout 36 days.

### Ovariectomy (OVX) and CUMS

For OVX surgery, mice were anesthetized with a mixture of ketamine (10 mg/kg) and xylazine (10 mg/kg) *via* intraperitoneal injection (i.p.). Following small bilateral dorsal flank incisions, bilateral ovaries were removed immediately. An additional group of mice who received sham surgery with similar incisions but without ovary removal served as control.

At 2 weeks after surgery, OVX mice received CUMS for 14 days with various types of mild stressors, including tail clamping for 1 min, water deprivation for 15 h, food deprivation for 15 h, restraint in a plastic tube for 4 h, cage tilting at 45 degree for 15 h, empty cage without nesting for 15 h, illumination in dark phase, and wet bedding (50 g sawdust/200 ml water) for 15 h. The animals received only one stressor per day and the same stressor was applied for no more than 2 consecutive days such that animals could not predict the occurrence of future stressor events. Our previous studies have confirmed that the addition of CUMS could better mimic distress experienced during menopause transition (16, 17).

### Drug Preparation and Treatment

There were five treatment groups. They were OVX+CUMS mice who received a treatment with vehicle, 0.3 mg/kg 17β-estradiol (E_2_), 2.5 g/kg JYP, or 5 g/kg JYP. The control group with sham surgery received vehicle treatment. All agents were delivered *via* oral gavage on a daily basis for 36 days.

For estrogen treatment, E_2_ (Sigma-Aldrich, St. Louis, MO) was prepared in stock ethanol solution (20 mg/ml) and then diluted with distilled water to a concentration of 0.03 mg/ml, at which the ethanol concentration of 0.15% yielded served as vehicle. OVX+CUMS mice were treated with 0.3 mg/kg E_2_ in a volume of roughly 0.2 ml vehicle. The dose of 0.3 mg/kg E2 was an optimal dose that can well mimic biochemical effects of estrogen therapy based on previous dose-dependent studies (24, 25).

JYP has been approved for marketing in China in 2002. JYP used in this study was generously provided by Henan Taifeng Biotechnology Co., Ltd (Zhengzhou, China) (manufacturer batch number: 20170901). The detailed manufacturing procedure of JYP was carried out in strict compliance with Good Manufacturing Practice (GMP) (https://patentimages.storage.googleapis.com/68/b2/47/ed1207e1332697/CN1404864A.pdf). The quality control of JYP adhered to the specifications and test procedures according to the internal standard. For animal treatment, JYP was dissolved in the vehicle and administered at 30 min before the CUMS stressors. The two doses of JYP (2.5 g/kg and 5 g/kg) used in mice were based on a clinically recommended dose of 12 gram per day for adults weighing 60 kg.

### Behavioral Tests

Anxiety-like behavior was examined in open-field test (OFT) and elevated plus maze (EPM). Depression-like behavior was examined using sucrose preference test (SPT), forced-swimming test (FST), and tail suspension test (TST). Cognitive performance was measured using Morris water maze.

#### Open-Field Test (OFT)

The test was conducted in a white square box (40 × 40 × 40 cm), in which the white floor is divided into the central zone (15 × 15 cm) and the surrounding zone. Each mouse was placed on the surrounding zone and allowed to explore freely for 5 min under white fluorescent light from above. Its movement trajectory in the box was recorded with video tracking software (Noldus Information Technology, Leesburg, VA, USA). The total distance moved was analyzed to evaluate locomotor activity. The time spent in and number of entries into the central zone were obtained to evaluate the extent of anxiety. The box was cleaned with 70% ethanol between tests.

#### Elevated Plus Maze (EPM)

EPM consists of two open arms (35 × 5 cm) and two closed arms (35 × 5 × 15 cm), arranged such that the two arms of each type are opposite to each other. The maze is elevated to a height of 70 cm from the floor. The mouse was placed in the center area of the maze with its head directed toward an open arm and was then allowed to explore the maze freely for 5 min. The movement trajectory in the maze was recorded with video tracking software (Noldus Information Technology, Leesburg, VA, USA). Any subsequent visit to one of the four arms was counted when all four paws of a mouse entered. The time spent in and number of entries into the open arms were obtained to evaluate the extent of anxiety. The maze was cleaned with 70% ethanol between tests.

#### Sucrose Preference Test (SPT)

To acclimatize sucrose preference, mice were exposed to two bottles containing 1% sucrose solution (w/v) with *ad libitum* access for 24 h in groups of three to five per cage. On day 2, one bottle containing 1% sucrose solution and another containing tap water were accessible for 24 h. On day 3, the position of the two bottles was switched for 24 h. At the end of the adaptation period, mice were deprived from food and water for 22 h. After that, SPT was conducted in an individual mouse housed in a cage with free access to two respective bottles containing 1% sucrose solution and tap water for 2 h. To prevent side preference in drinking behavior, the position of the two bottles was switched in the middle of testing. Water and sucrose consumption were measured as changes in weight of fluid consumed. The sucrose preference was calculated from the following formula: sucrose preference (%) = sucrose intake (g)/[sucrose intake (g) + water intake (g)] × 100%.

#### Forced-Swimming Test (FST)

A polycarbonate cylinder (30 cm in height and 20 cm in diameter) was used for FST. The cylinder was filled with water to a depth of 15 cm at 23 to 25°C. A mouse was placed in the cylinder for 6 min and its movement was recorded on videotape. Immobility time, defined as the absence of all movements except for motions required to maintain the head above the water, was obtained from the last 4 min of the trial with EthoVision XT7 software.

#### Tail Suspension Test (TST)

The test was conducted in a specially manufactured tail suspension box. Each mouse was suspended 50 cm above the floor of the box by fixing its tail tip (1 cm in length) with adhesive tape. Immobility time, defined as the absence of any movements of limbs and trunk except for whisker movement and respiration, was recorded on videotape over 6 min of testing and analyzed with EthoVision XT7 software.

#### Morris Water Maze (MWM) Test

The apparatus for MWM consists of an open circular pool (90 cm in diameter and 45 cm in height) filled with water (22 ± 2°C). The pool was conceptually divided into four quadrants, and a hidden platform (6 cm in diameter and 39 cm in height) was placed in one of the quadrants and submerged 1 cm beneath the water surface. The test was divided into the two phases: 6 days of acquisition trials and 1 day of probe trial.

For acquisition trials, at Day 1 the mice were allowed to freely swim in clear water with the visual cues on the hidden platform. During the subsequent 5 days, the mice were given four training trials per day in the milky water by placing titanium dioxide into the water. Different starting points were used on each of the five daily trials and the order of starting point was random. If a mouse located the platform, it was allowed to remain on the platform for 10 s. If a mouse failed to locate the platform within 60 s, it was placed on the platform for 10 s. The mice were towel-dried and returned to their home cage after each trial. The latency to the hidden platform was recorded from each training trial and averaged across the four training trials each day for data analysis. One mouse of the 5 JYP group died at Day 2 during the acquisition trials.

The probe trial was conducted at Day 7. The platform was removed from the pool and a mouse was placed onto the pool and allowed to search the platform for 60 s. The movement trajectory in the maze was recorded with video tracking software (Noldus Information Technology, Leesburg, VA, USA). The total swimming distance was calculated to evaluate the locomotor activity. The latency to the target zone, the duration spent in the target zone, and the crossing number into the target zone were obtained to measure the spatial memory.

### Tissue Preparation and Measurement of Serum HPO- and HPA-Related Hormones

Following the completion of behavioral tests, animals were anesthetized with ketamine/xylazine (120/18 mg/kg). A volume of 0.5–0.6 ml blood was collected *via* cardiac puncture. The uterus and whole brains were removed. The prefrontal cortex, hippocampus, and hypothalamus were dissected from the brains for Western blot analysis (see below). Sera were immediately separated by centrifuging at 3,500 rpm for 15 min at 4°C and stored at −80°C until assay. Adrenocorticotropic hormone (ACTH) (Cat No.: RK02566), Follicle Stimulating Hormone (FSH) (Cat No.: RK02819) and Luteinizing Hormone (LH) (Cat No.: RK02986) levels were measured using their corresponding commercial enzyme-linked immunosorbent assay (ELISA) kits (ABclonal Technology, Wuhan, China) according to the manufacturer's instructions. Estradiol (E_2_) (Cat No.: KGE014) and Corticosterone (CORT) (Cat No.: KGE009) were also tested with ELISA kits (R&D systems Inc., Minneapolis, USA).

### Determination of Brain Regional GABA and Glutamate Levels

High performance liquid chromatography (HPLC) with diode-array detector (DAD) was used to measure the contents of regional brain GABA and glutamate (Glu) as described previously (26). Briefly, the hypothalamus, hippocampus, and prefrontal cortex were dissected from the brain and homogenized in 100–200 μl of acetonitrile. The homogenate was centrifuged at 13,000 rpm at 4°C for 20 min; the supernatant was collected and evaporated under a gentle stream of nitrogen. The dried residue was reconstituted in 50–100 μl of Na_2_CO_3_-NaHCO_3_ buffer (pH 9.5). The 25–50 μl of dansyl chloride (10 mmol/L) was added and vortex mixed for derivatization procedure, and the mixture was incubated in dark at 65°C for 25 min and cooled to room temperature. The solution was then centrifuged at 13,000 rpm for 20 min, and 10 μl of the supernatant was directly injected into a Thermo 3000 series UPLC equipped with an ACE Excel 2 C18 column (100 mm × 2.1 mm × 1.7 μm) and DAD. The mobile phase consisted of acetonitrile (A) and 0.6% acetic acid in water/0.008% trithylamine (B). The gradient program was developed with 70–55% B for 0–20 min. The flow rate was kept at 0.4 mL/min and the detective wavelength was selected at 254 nm.

### Western Blot Analysis

Western blot analysis was used to detect the effects of JYP on the expression levels of N-methyl-D-aspartate receptor subunit 1 (NMDAR1), the three neurotrophins, brain-derived neurotrophic factor (BDNF), nerve growth factor (NGF), and glial cell-derived neurotrophic factor (GDNF), and the two estrogen receptors, ERα and ERβ, in the brain regions and the uterus. Brain and uterine tissues were homogenized in radio-immunoprecipitation assay buffer (RIPA Buffer, Sigma Aldrich, USA) containing 2% phenylmethanesulfonyl fluoride (PMSF, Sigma-Aldrich, USA) at 4°C for 30 min. All tissues were centrifuged at 13,000 rpm for 20 min. The supernatant was collected and their protein concentration were measured with the Bradford method using Coomassie brilliant blue G-250 (Bio-Rad Laboratories Inc.). Proteins were separated by electrophoresis on 10% SDS-PAGE gels and subsequently transferred onto polyvinylidene difluoride membranes (PVDF, 0.22 μM, Bio-Rad Laboratories, Inc.). After being blocked with 5% BSA in TBST, the membranes were then blotted with the primary rabbit antibodies against NMDAR1 (1:2000, Santa Cruz Biotechnology, USA, Cat No.: sc-1468), BDNF (1:1000, Santa Cruz Biotechnology, USA, Cat No.: sc-546), NGF (1:1000, Abcam, Cambridge, USA, Cat No.: ab52918), GDNF (1:1000, Abcam, Cambridge, USA, Cat No.: ab176564), ERα (1:1000, invitrogen, USA, Cat No.: PA5-16440), ERβ (1:1000, invitrogen, USA, Cat No.: PA1-310B), and mouse anti-GAPDH (1:5000, Immunoway, USA, Cat No.:YM3029) at 4°C overnight. After rinsing with TBST, the membranes were incubated with suitable secondary antibodies (1:2000, Santa Cruz Biotechnology, USA) at 4°C for 4 h. Chemiluminescence was detected using an enhanced chemiluminescence detection kit (GE Healthcare, UK). The intensity of the bands was quantified by scanning densitometry using Image Lab 5.1 software (Bio-Rad, Laboratories, Inc.). The mean value of the intensity was obtained from at least three independent experiments.

### Statistical Analysis

Based on our previous studies (16, 17), *n* = 10 per group should be sufficient to detect statistical differences in behavioral variables among treatment groups at a power of 0.8 and a significance level of 0.05. Two-way repeated measure analysis of variance (ANOVA) was used to detect the effects of JYP on the latency to the hidden platform over acquisition trials. One-way ANOVA was used to examine other variables. Between-group differences were further analyzed using Student–Newman–Keuls test. All data were expressed as mean ± standard error of the mean (SEM). Statistical significance was defined as <0.05 of *P-*value. All statistical analysis was conducted with SAS (version 9.4; SAS Institute, Cary, NC).

## Results

### Effects of JYP and E_2_ on Body Weight and Uterine Weight

**Figure 2:**
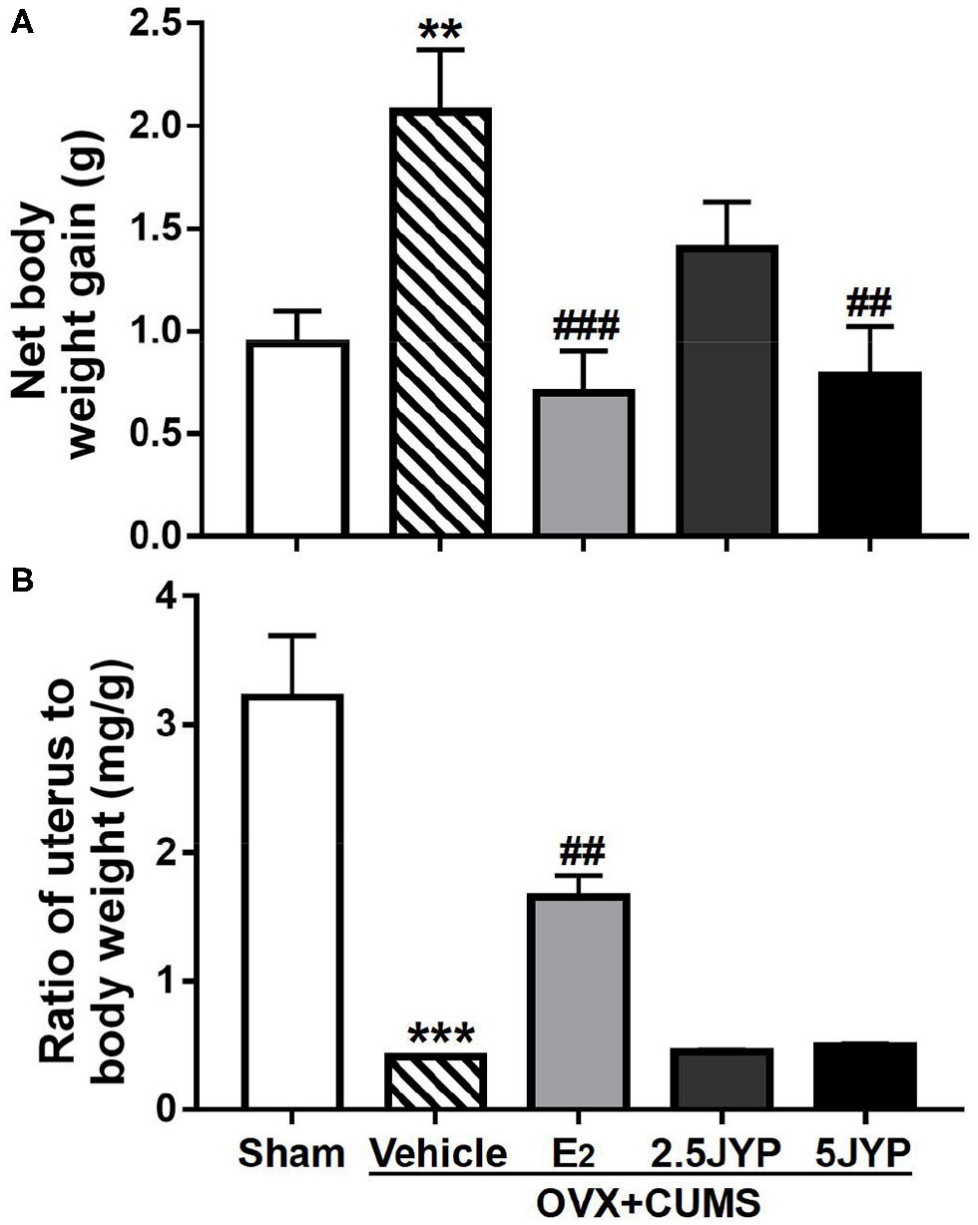
Effects of 2.5 g/kg JYP (2.5 JYP), 5.0 g/kg JYP (5 JYP), and 0.3 mg/kg estradiol (E2) on net changes in body weight **(A)** and ratio of uterus to body weight **(B)** of OVX+CUMS mice. Data are expressed as mean ± SEM (*n* = 9–10) and examined with one-way analysis of variance (ANOVA), followed by *post hoc* between-group comparisons: **P < 0.01, ***P < 0.001 vs. sham group; ^##^*P* < 0.01, ^###^*P* < 0.001 vs. vehicle group.

Significant effects of group were observed on body weight [*F*_(4, 44)_ = 6.088, *P* < 0.001] and ratio of uterus to body weight [*F*_(4, 44)_ = 27.6, *P* < 0.001] (Figure 2). OVX+CUMS caused a striking increase in body weight (*P* = 0.004) (Figure 2A) and a dramatic decrease in ratio of uterus to body weight (*P* < 0.001) compared to sham surgery (Figure 2B). E_2_ treatment partially reversed OVX+CUMS-induced changes in the two variables (*P* ≤ 0.002). JYP (5 g/day) significantly suppressed the OVX+CUMS-induced weight gain (*P* = 0.001), but either dose of JYP had no effects on the ratio of uterus to body weight compared to vehicle (*P* ≥ 0.997).

### Effects of JYP and E_2_ on Anxiety-Like Behavior

**Figure 3:**
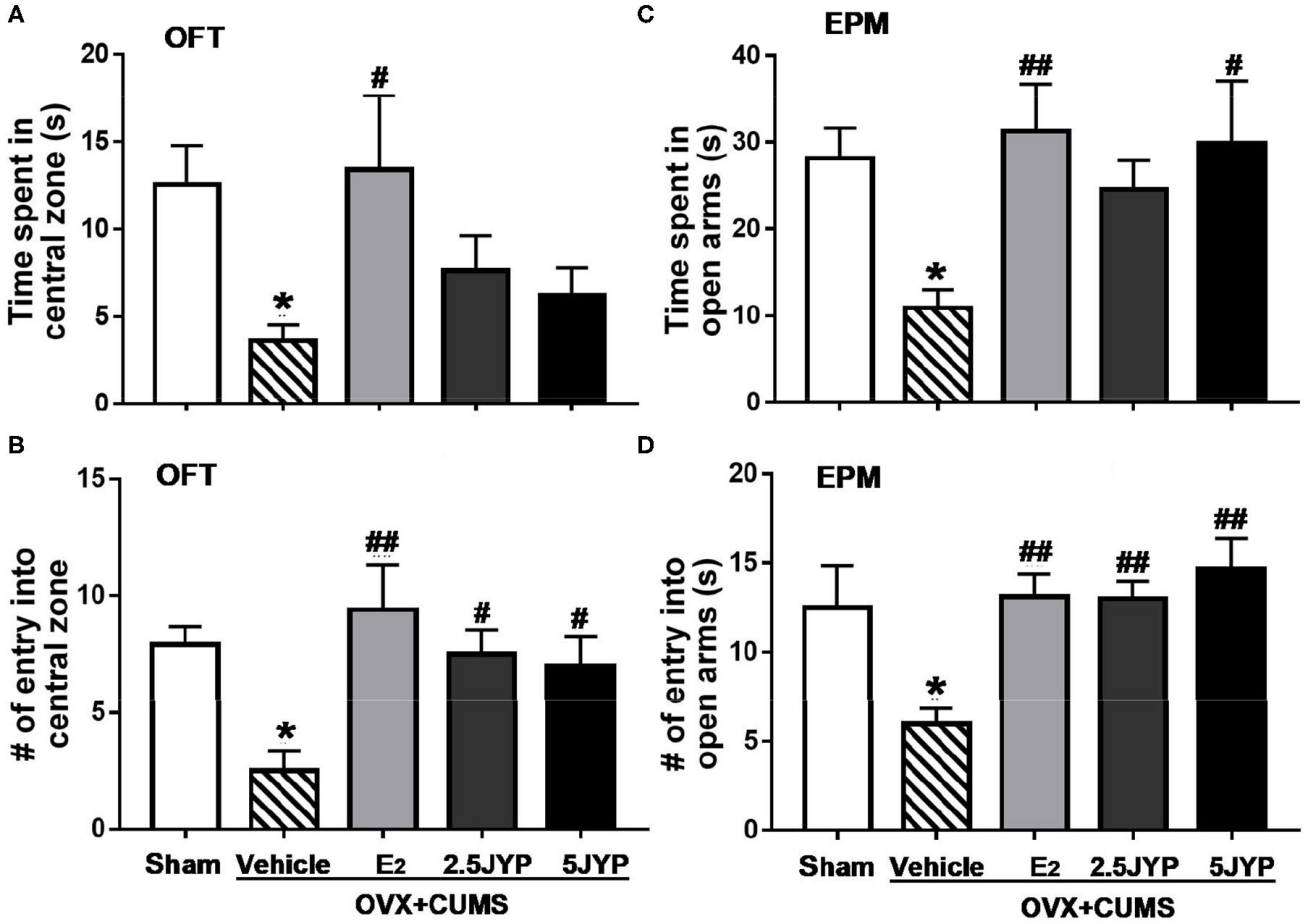
Effects of 2.5 g/kg JYP (2.5 JYP), 5.0 g/kg JYP (5 JYP), and 0.3 mg/kg estradiol (E2) on OVX+CUMS-induced anxiety-like behavior: **(A)** Duration in the central zone in the open field test (OFT); **(B)** Number of entries into the central zone in the OFT; **(C)** Duration in the open arms in the elevated plus maze (EPM) test; and **(D)** Number of entries into the open arms in EPM. Data are expressed as mean ± SEM (*n* = 9–10) and examined with one-way analysis of variance (ANOVA), followed by *post hoc* between-group comparisons: *P < 0.05 vs. sham group; ^#^*P* < 0.05, ^##^*P* < 0.01 vs. vehicle group.

There were significant differences among the four groups in time spent in [*F*_(4, 44)_ = 2.754, *P* = 0.037] and number of entries into the central zone of OFT [*F*_(4, 44)_ = 4.388, *P* = 0.005], and time spent in [*F*_(4, 44)_ = 3.35, *P* = 0.018] and number of entries into open arms of EPM [*F*_(4, 44)_ = 4.828, *P* = 0.003] (Figure 3). In OFT, OVX+CUMS mice treated with E_2_ spent markedly more time in and had more entries into the central zone (*P* ≤ 0.027) compared to those treated with vehicle (Figures 3A,B). Both doses of JYP significantly increased the entry number to the central zone (*P* ≤ 0.031). In EPM, OVX+CUMS mice treated with E_2_ and 5 g/kg JYP spent significantly more time in and had more entries into open arms than those treated with vehicle (*P* ≤ 0.020) (Figures 3C,D). OVX+CUMS mice receiving 2.5 g/kg JYP also showed significantly more entries into open arms than those vehicle-treated OVX+CUMS mice (*P* = 0.008).

### Effects of JYP and E_2_ on Depression-Like Behavior

**Figure 4:**
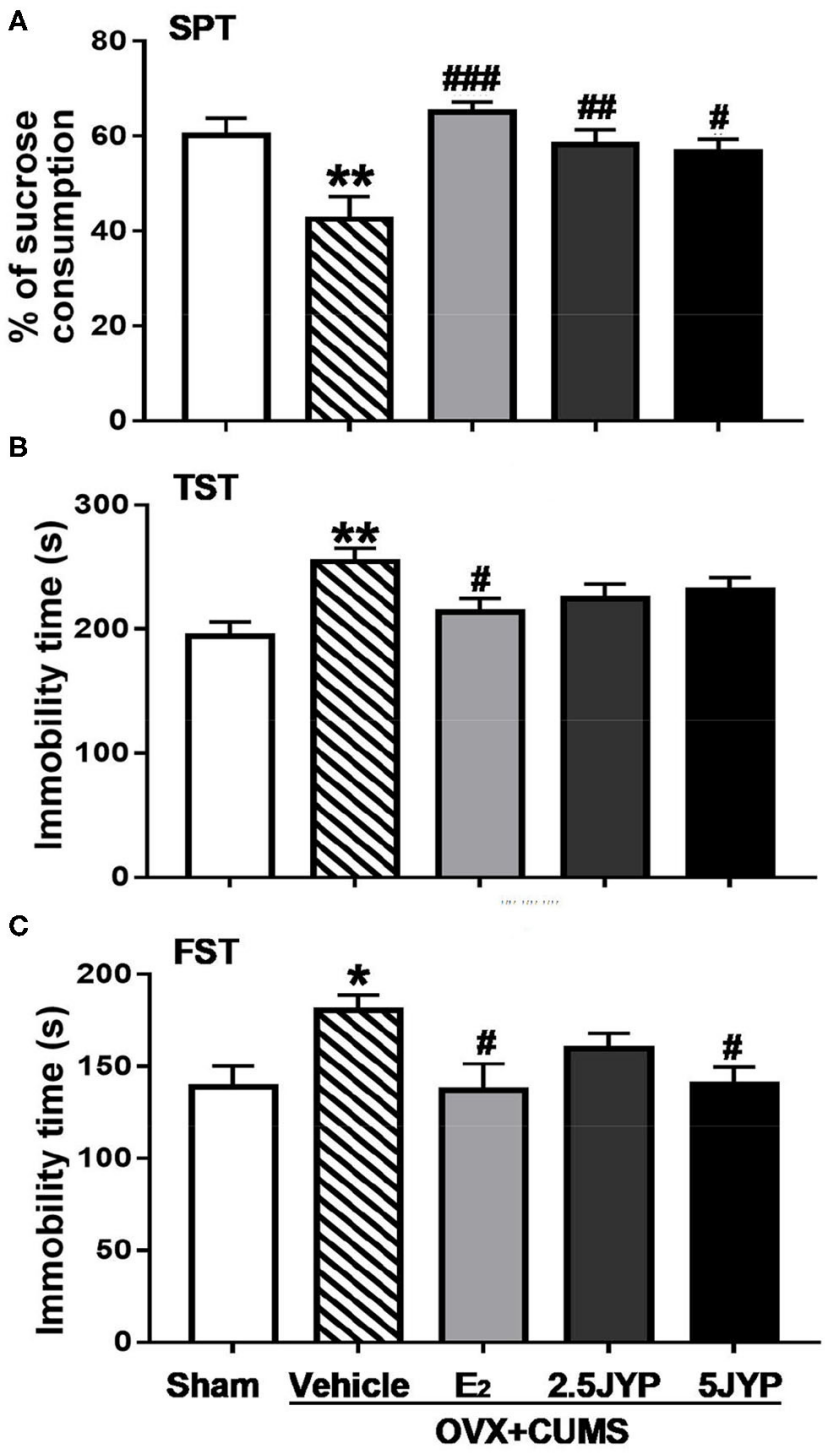
Effects of 2.5 g/kg JYP (2.5 JYP), 5.0 g/kg JYP (5 JYP), and 0.3 mg/kg estradiol (E2) on OVX+CUMS-induced depression-like behavior: **(A)** percentage of sucrose consumption in sucrose preference test (SPT); **(B)** immobility time of tail suspension test (TST); and **(C)** immobility time of forced swimming test (FST). Data are expressed as mean ± SEM (*n* = 9–10) and examined with one-way analysis of variance (ANOVA), followed by *post hoc* between-group comparisons: *P < 0.05, **P < 0.01 vs. sham group; ^#^*P* < 0.05, ^##^*P* < 0.01, ^###^*P* < 0.001 vs. vehicle group.

Significant effects of group were present on sucrose consumption in SPT [*F*_(4, 44)_ = 6.221, *P* < 0.001] (Figure 4A), immobility time in TST [*F*_(4, 44)_ = 4.01, *P* = 0.007] (Figure 4B), and FST [*F*_(4, 44)_ = 3.138, *P* = 0.024] (Figure 4C). OVX+CUMS resulted in a marked reduction of sucrose consumption in SPT and a significant increase in immobility time spent in TST and FST compared to sham surgery (*P* ≤ 0.026). E_2_ completely reversed OVX+CUMS-induced changes in the three variables (*P* ≤ 0.045). Both doses of JYP significantly increased sucrose consumption (*P* ≤ 0.023) and 5 g/kg JYP additionally reduced immobility time spent in FST (*P* < 0.039) as compared to vehicle.

### Effects of JYP and E_2_ on Cognitive Performance

**Figure 5:**
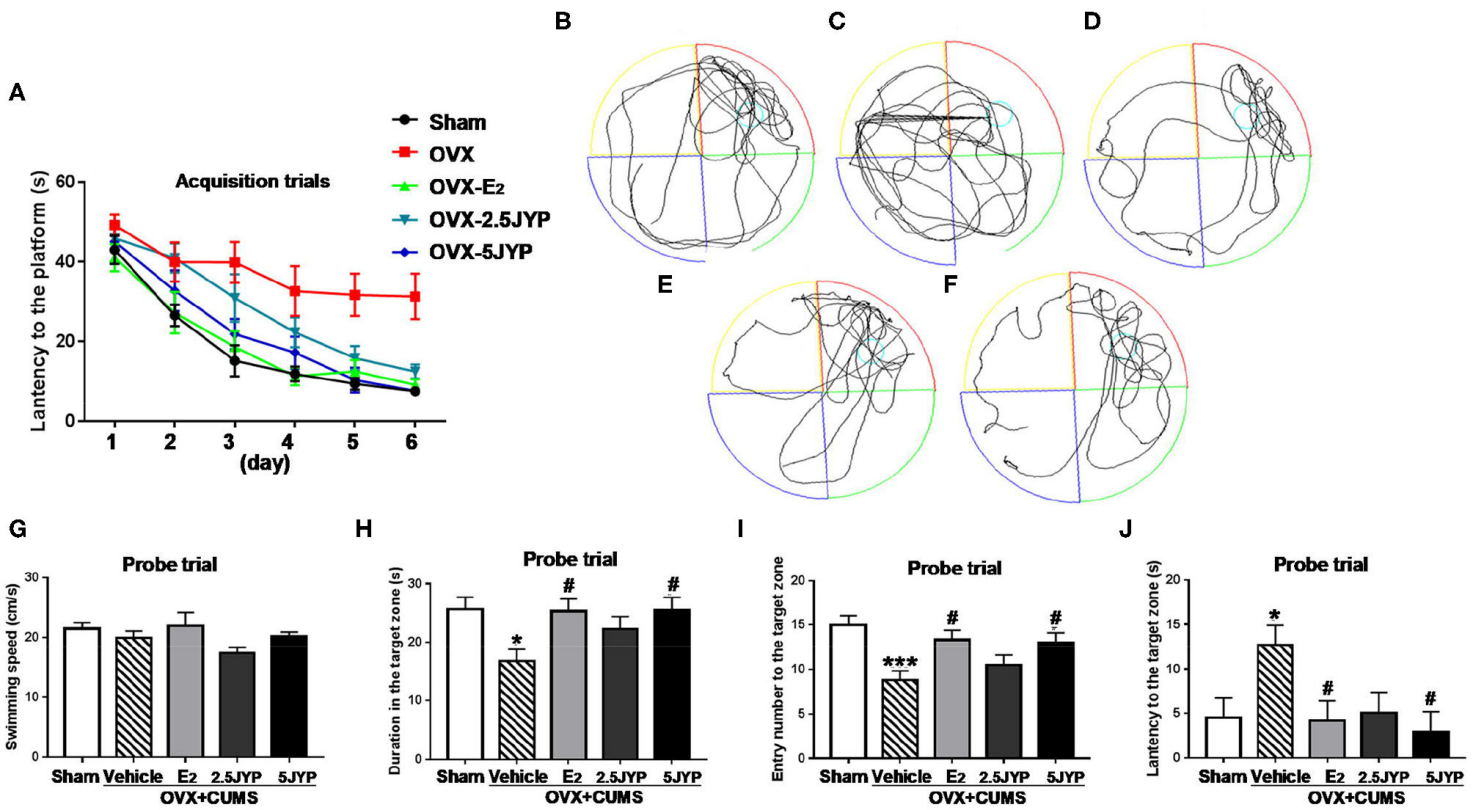
Effects of 2.5 g/kg JYP (2.5 JYP), 5.0 g/kg JYP (5 JYP), and 0.3 mg/kg estradiol (E2) in Morris water maze test of OVX+CUMS mice: escape latency in the acquisition trials **(A)**; representative individual swim paths in the probe trial of sham group **(B)**, vehicle group **(C)**, E_2_ group **(D)**, 2.5 JYP group **(E)**, and 5 JYP group **(F)**; swimming speed **(G)**, duration in the target zone **(H,I)** entry number into the target zone **(I)**, and latency to the target zone **(J)** in the probe trial. Data are expressed as mean ± SEM (*n* = 9–10) and examined with two-way or one-way analysis of variance (ANOVA) and one-way ANOVA, followed by followed by *post hoc* between-group comparisons: *P < 0.05, ***P < 0.001 vs. sham group; ^#^*P* < 0.05 vs. vehicle group.

Two-way ANOVA showed no significant interaction between group and time on the escape latency to the platform [*F*_(20, 220)_ = 1.300, *P* = 0.203], but significant main effects were observed on group [*F*_(4, 44)_ = 11.10, *P* < 0.001] and time [*F*_(5, 220)_ = 41.61, *P* < 0.001] on the latency in acquisition trials (Figure 5A). Vehicle-treated OVX+CUMS mice spent much longer latency to find the platform than mice with sham surgery at Day 2 through Day 6 (*P* ≤ 0.033). The latency of OVX+CUMS mice treated with E_2_ was markedly less than that of OVX+CUMS mice treated with vehicle at Day 2 through Day 6 (*P* ≤ 0.040). OVX+CUMS mice treated with 2.5 g/kg JYP spent less time locating the platform at Day 5 and Day 6 (*P* ≤ 0.009). OVX+CUMS mice treated with 5 g/kg JYP showed a shorter latency to find the platform at Day 3 through Day 6 (*P* ≤ 0.016).

In the probe trial, representative individual swim paths from each group are shown in Figures 5B–F. Treatment had no effects on swimming speed [*F*_(4, 44)_ = 1.755, *P* = 0.155] (Figure 5G), but significantly changed time spent in [*F*_(4, 44)_ = 3.278, *P* = 0.020] (Figure 5H), number of entries into [*F*_(4, 44)_ = 5.142, *P* = 0.002] (Figure 5I), and latency to the target zone [*F*_(4, 44)_ = 3.000, *P* = 0.028] (Figure 5J). OVX+CUMS markedly reduced time stayed in (*P* = 0.016) and number of entries into the target zone (*P* < 0.001), and increased the latency to the target zone (*P* = 0.044) compared to sham surgery. OVX+CUMS mice treated with E_2_ and 5 g/kg JYP remarkably increased time spent in (*P* ≤ 0.022), frequency crossed (*P* ≤ 0.035), and shorter latency to the target zone (*P* ≤ 0.034) compared to those treated with vehicle.

### Effects of JYP and E_2_ on HPO-and HPA-Related Hormones

**Figure 6:**
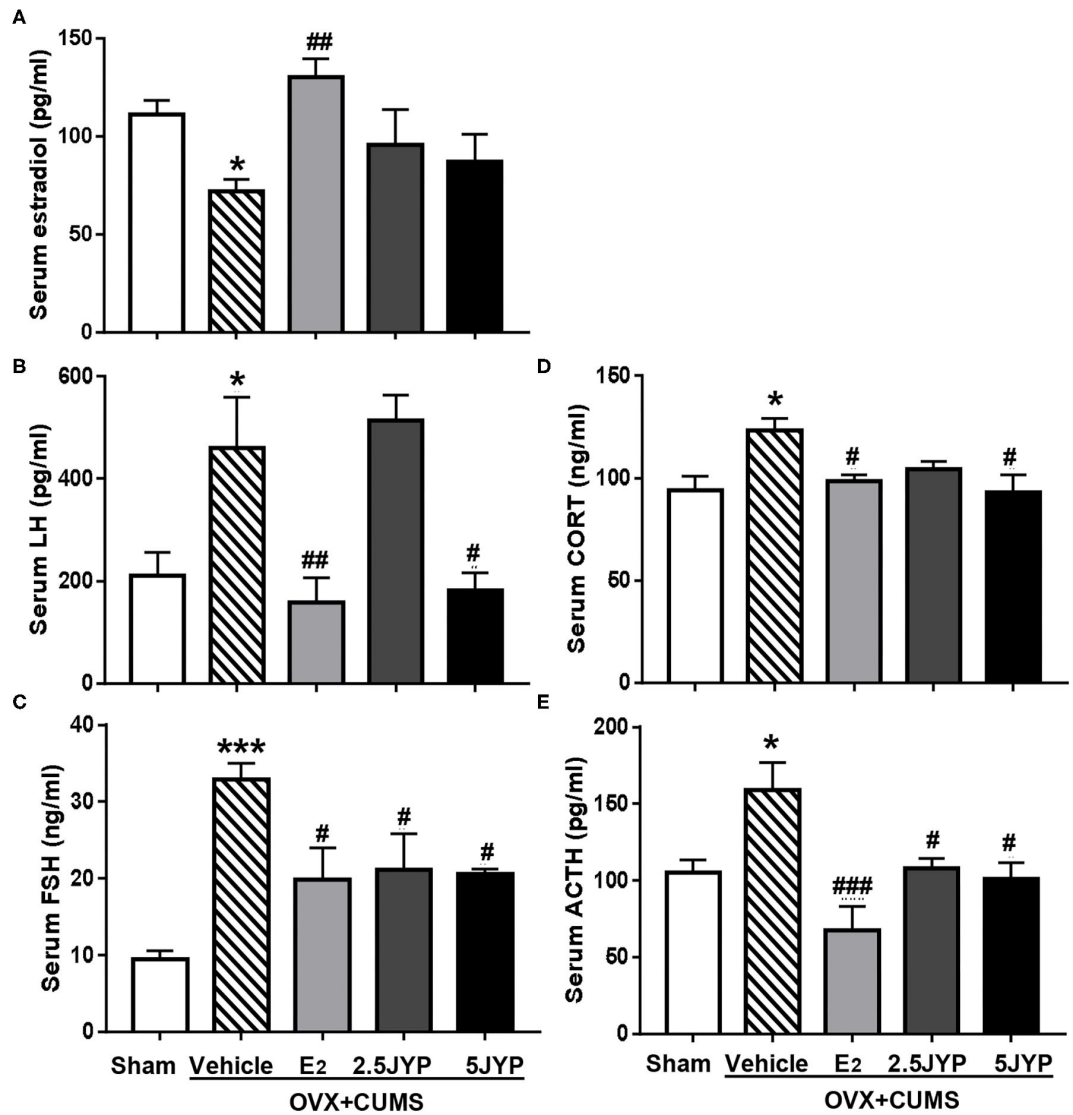
Effects of 2.5 g/kg JYP (2.5 JYP), 5.0 g/kg JYP (5 JYP), and 0.3 mg/kg estradiol (E2) on serum hypothalamic–pituitary–adrenal (HPA) and hypothalamic-pituitary-ovarian (HPO)-axis hormones with E_2_ **(A)**, luteinizing hormone (LH) **(B)**, follicle stimulating hormone (FSH) **(C)**, corticosterone (CORT) **(D)**, and adrenocorticotropic hormone (ACTH) **(E)**. Data are expressed as mean ± SEM (*n* = 4) and examined with one-way analysis of variance (ANOVA), followed by *post hoc* between-group comparisons: *P < 0.05, ***P < 0.001 vs. sham group; ^#^*P* < 0.05, ^##^*P* < 0.01, ^###^*P* < 0.001 vs. vehicle group.

Significant main effects of groups were observed on serum E_2_ (Figure 6A), FSH (Figure 6B), and LH [*F*_(4, 15)_ ≥ 5.543, *P* ≤ 0.006] (Figure 6C). OVX+CUMS strikingly decreased serum level of E_2_ and increased FSH and LH levels compared with sham surgery (*P* ≤ 0.033). E_2_ treatment completely reversed OVX-induced changes in levels of the three hormones. Both doses of JYP significantly suppressed the elevated levels of FSH (*P* ≤ 0.045); 5 g/kg JYP additionally suppressed the OVX+CUMS-induced elevation of LH level (*P* = 0.017).

Significant differences were observed in serum level of CORT (Figure 6D) and ACTH (Figure 6E) across the five groups [*F*_(4, 15)_ ≥ 4.059, *P* ≤ 0.020]. OVX+CUMS resulted in a significant elevation of the two hormones (*P* ≤ 0.028), which were completely reversed by E_2_ and 5 g/kg JYP. JYP at 2.5 g/kg also completely reversed OVX+CUMS-induced elevation of ACTH level (*P* ≤ 0.038).

### Effects of JYP and E_2_ on Brain Regional Contents of GABA and Glutamate

**Figure 7:**
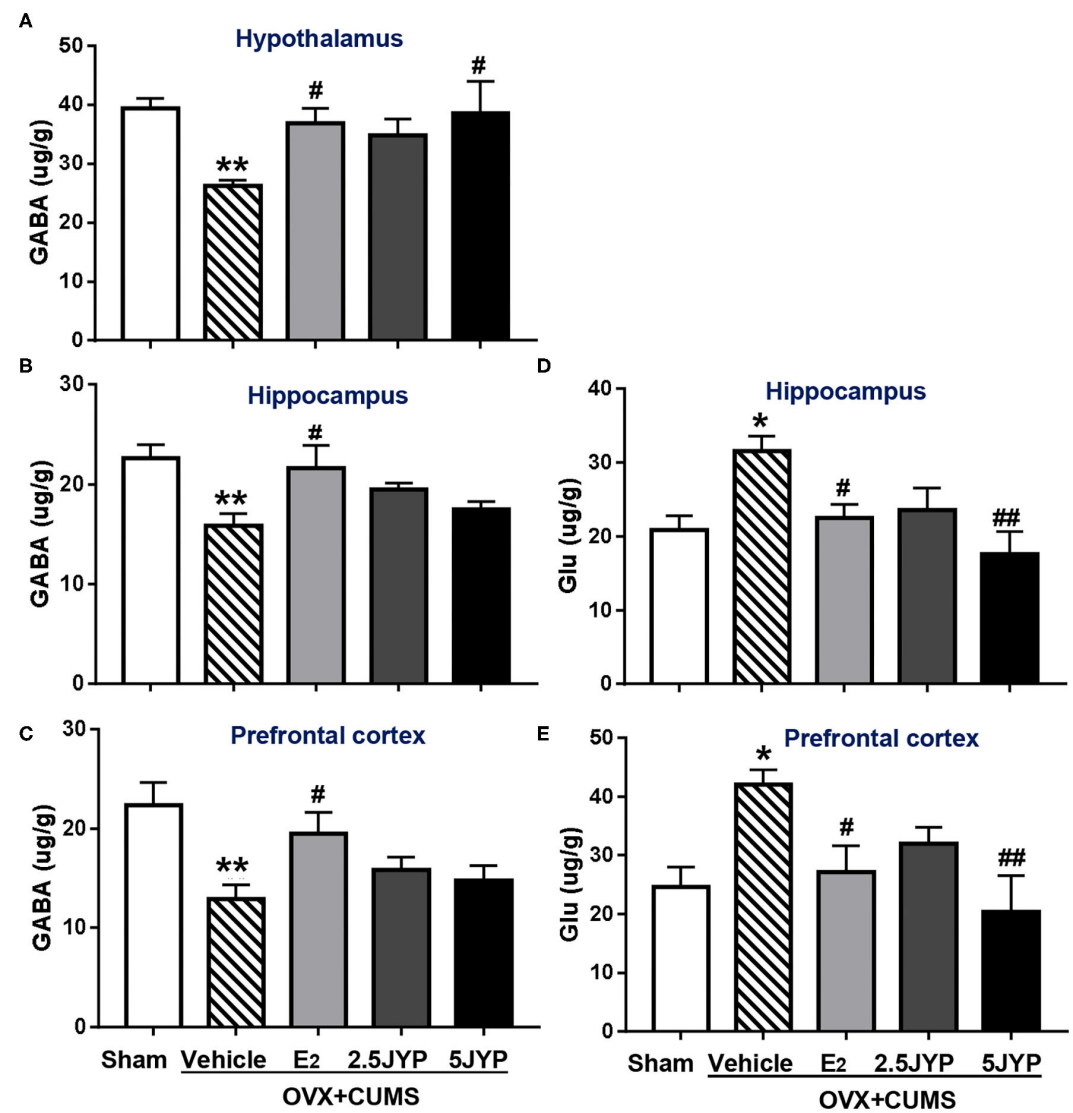
Effects of 2.5 g/kg JYP (2.5 JYP), 5.0 g/kg JYP (5 JYP), and 0.3 mg/kg estradiol (E2) on brain regional γ-Aminobutyric acid (GABA) and glutamate (Glu) contents. **(A)** GABA in hypothalamus; **(B)** GABA in hippocampus; **(C)** GABA in prefrontal cortex; **(D)** Glu in hippocampus; **(E)** Glu in prefrontal cortex. Data are expressed as mean ± SEM (*n* = 5–6) and examined with one-way analysis of variance (ANOVA), followed by *post hoc* between-group comparisons: *P < 0.05, **P < 0.01 vs. sham group; ^#^*P* < 0.05, ^##^*P* < 0.01 vs. vehicle group.

There were significant differences in the contents of GABA and glutamate in the hypothalamus (Figure 7A), hippocampus (Figures 7B,D), and prefrontal cortex (Figures 7C,E), among groups [*F*_(4, 24)_ ≥ 3.544, *P* ≤ 0.021]. OVX+CUMS mice showed marked decreases of GABA in the three brain regions (*P* ≤ 0.010), which were significantly restored by E_2_ treatment (*P* ≤ 0.046). The high dose of JYP also restored the decrease of hypothalamic GABA (*P* ≤ 0.023). The contents of hippocampal and prefrontal glutamate of OVX+CUMS mice were much higher than those with sham surgery (*P* ≤ 0.013). OVX+CUMS mice exposed to stress and treated with E_2_ and the high dose of JYP completely reversed the treatment (*P* ≤ 0.038). The contents of glutamate in the hypothalamus failed to be detected.

### Effects of JYP and E_2_ on Regional Brain NMDAR1

**Figure 8:**
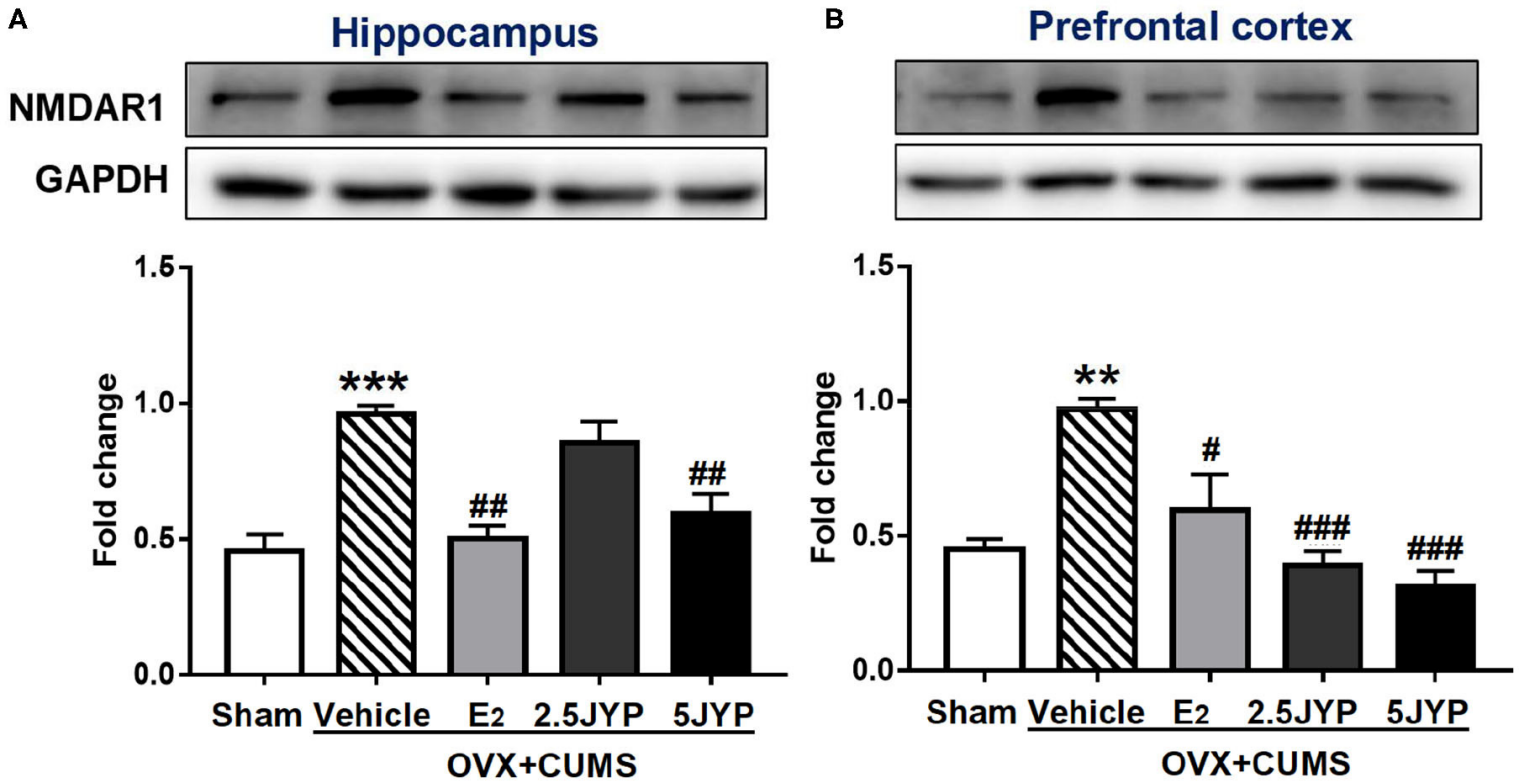
Effects of 2.5 g/kg JYP (2.5 JYP), 5.0 g/kg JYP (5 JYP), and 0.3 mg/kg estradiol (E2) on the expression of N-methyl-D-aspartate (NMDA) receptor unit 1 (NMDAR1) in the hippocampus **(A)** and prefrontal cortex **(B)**. Data are expressed as mean ± SEM (*n* = 3) and examined with one-way analysis of variance (ANOVA), followed by *post hoc* between-group comparisons: **P < 0.01, ***P < 0.001 vs. sham group; ^#^*P* < 0.05, ^##^*P* < 0.01, ^###^*P* < 0.001 vs. vehicle group.

Significant group effects were detected on the expression of NMDAR1 in the hippocampus (Figure 8A) and prefrontal cortex (Figure 8B) [*F*_(4, 10)_ ≥ 12.51, *P* ≤ 0.001]. The expression levels of NMDAR1 in both brain regions of OVX+CUMS mice were ~2-fold higher than those with sham surgery (*P* ≤ 0.002), but completely reversed by treatment with E_2_ and the high dose JYP (*P* ≤ 0.016). The low dose JYP also strikingly suppressed the OVX+CUMS-induced increase of prefrontal NMDAR1 expression (*P* < 0.001).

### Effects of JYP and E_2_ on Brain Regional and Uterine Neurotrophins

**Figure 9:**
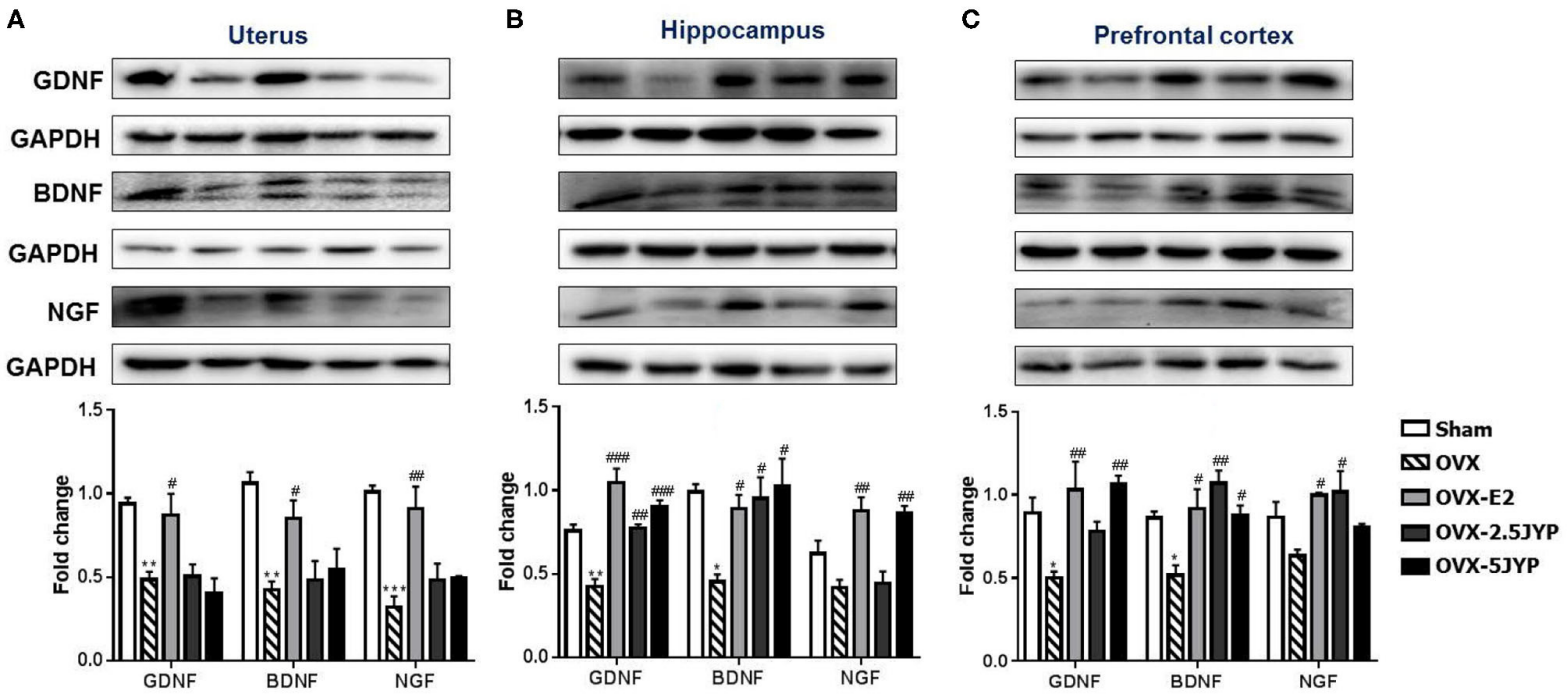
Effects of 2.5 g/kg JYP (2.5 JYP), 5.0 g/kg JYP (5 JYP), and 0.3 mg/kg estradiol (E2) on the expression of glial cell-derived neurotrophic factor (GDNF), brain-derived neurotrophic factor (BDNF), and nerve growth factor (NGF) in the uterus **(A)**, hippocampus **(B)**, and prefrontal cortex **(C)**. Data are expressed as mean ± SEM (*n* = 3) and examined using one-way analysis of variance (ANOVA), followed by *post hoc* between-group comparisons: *P < 0.05, **P < 0.01, vs. sham group; ^#^*P* < 0.05, ^##^*P* < 0.01, ^###^*P* < 0.001 vs. vehicle group.

GDNF, BDNF, and NGF expression levels significantly differed among groups in the uterus (Figure 9A), hippocampus (Figure 9B), and prefrontal cortex (Figure 9C) [*F*_(4, 10)_ ≥ 4.691, *P* ≤ 0.022] OVX+CUMS dramatically suppressed the expression of the three neurotrophins in the three tissues (*P* ≤ 0.046) except for hippocampal and prefrontal NGF compared to sham surgery. E_2_ completely restored all the three neurotrophin expression in the three tissues examined (*P* ≤ 0.046) compared to vehicle treatment. Both doses of JYP also completely reversed the OVX+CUMS-induced decreases of hippocampal GDNF and BDNF and prefrontal NGF (*P* ≤ 0.024), but had no significant effects on uterine neurotrophins. JYP-treated OVX+CUMS mice displayed strikingly higher expression levels of hippocampal NGF and prefrontal GDNF with the high dose (*P* ≤ 0.006) and prefrontal NGF (*P* = 0.012) with the low dose than those treated with vehicle.

### Effects of JYP and E_2_ on Brain Regional and Uterine Estrogen Receptors

**Figure 10:**
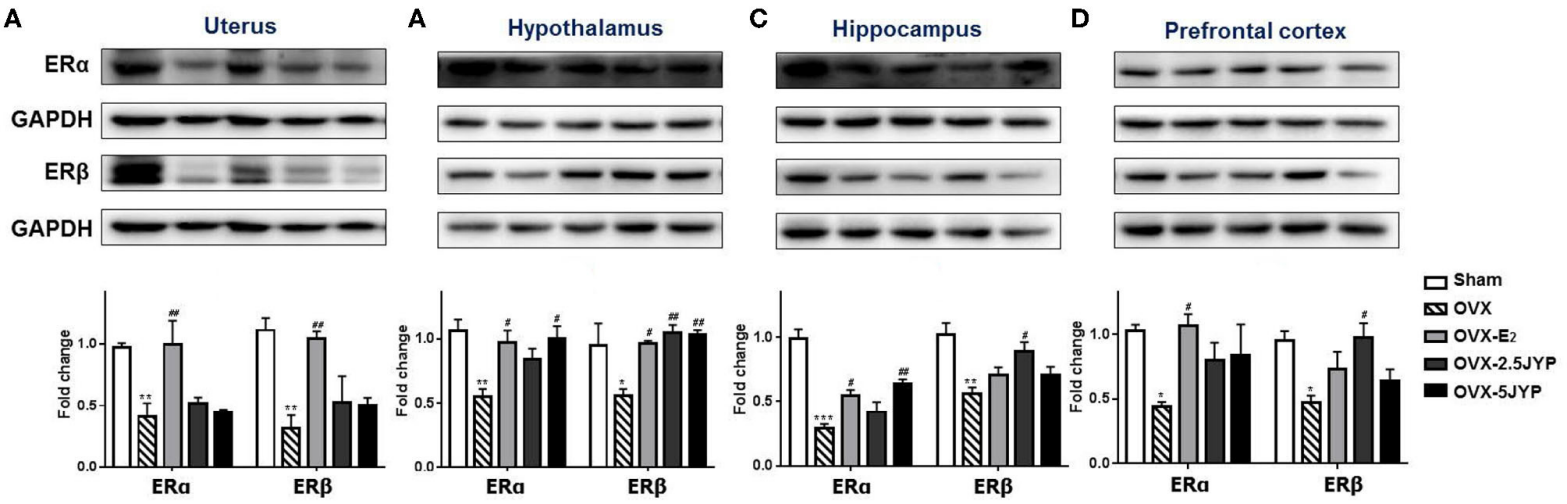
Effects of 2.5 g/kg JYP (2.5 JYP), 5.0 g/kg JYP (5 JYP), and 0.3 mg/kg estradiol (E2) on the expression of ERα and ERβ in the uterus **(A)**, hypothalamus **(B)**, hippocampus **(C)**, and prefrontal cortex **(D)**. Data are expressed as mean ± SEM (*n* = 3) and examined with one-way analysis of variance (ANOVA), followed by *post hoc* between-group comparisons: *P < 0.05, **P < 0.01, ***P < 0.001 vs. sham group; ^#^*P* < 0.05, ^##^*P* < 0.01, vs. vehicle group.

There were marked differences in the expression level of ERα and ERβ in the uterus (Figure 10A), hypothalamus (Figure 10B), hippocampus (Figure 10C), and prefrontal cortex (Figure 10D) among groups [*F*_(4, 10)_ ≥ 3.626, *P* ≤ 0.045]. OVX+CUMS markedly suppressed the expression of both receptor types in all the four tissues examined compared to sham surgery (*P* ≤ 0.031). E_2_ partially or completely restored the expression of the two receptor types in the four tissues compared to vehicle (*P* ≤ 0.030). Both doses of JYP significantly increased the expression level of hypothalamic ERβ (*P* ≤ 0.010), but had no effects on both receptor types in the uterus. The high dose JYP partially or completely restored the expression of hippocampal and hypothalamic ERα (*P* ≤ 0.013), and the low dose almost completely restored the expression level of hippocampal and prefrontal ERβ (*P* ≤ 0.021) compared to vehicle.

## Discussion

The main purpose of this study was to evaluate the therapeutic effects of JYP as a novel therapy in improving mood and cognitive symptoms associated with menopause. In previous studies, we found that OVX alone could not constantly evoke anxiety- and mood disorder-like behavior; the addition of CUMS exposure however augmented aberrant behaviors (16, 17). This combination model also has been well-validated in several recent studies (28–30). Indeed, in this study, OVX mice exposed to CUMS showed remarkable anxiety-like behavior, manifesting as marked decrease in time spent and number of entry in central zone of the open field and in open arms of EPM compared to the control group. OVX combined with CUMS also evoked depression-like behavior, with significant decrease in sucrose consumption and increase in immobility time in TST and FST. The combination of OVX and CUMS further impaired spatial learning and memory ability, evidenced by strikingly longer latency in finding the platform in training trials and in reaching the target zone in the probe trial, and less duration and number of entry in the target zone than the control group in water maze test. These results, once again, confirm that OVX mice with stress exposure is a valid model in mimicking estrogen deprivation-induced psychiatric disorders.

Treatment with JYP in particularly higher dose, however, suppressed anxiety- and depression-like behavior and prevent spatial learning and memory to a similar degree as did E_2_, suggesting comparable efficacy of JYP in the treatment of estrogen deprivation induced psychiatric disorders. This is also highly consistent with anxiolytic and antidepressant effects of JYP observed in male rodents with (10, 11) and without exposure to CUMS (7, 8). It therefore appears that JYP not only could reduce “generalized” anxiety and “endogenous” depression, but is also effective in alleviating stress-associated mood disorders.

This study further revealed that the OVX mice exposed to stress displayed large weight gain and uterine shrinkage. Weight gain and shrinkage of the urogenital organs are the two major physical changes occurring during menopausal transition (31, 32). While long-term repeated E_2_ treatment suppressed OVX+CUMS-induced weight gain, it also caused uterine hyperplasia that may be indicative of adenomyosis, uterine fibroids, ovarian cysts, and even endometrial cancer (33). Such side effect has been widely observed in hormone replacement therapy in menopause women (34). However, unlike E_2_, JYP, in either low or high dose, did not influence changes in either body or uterine weight induced by OVX combined with CUMS. It seems that JYP has minor or even no effects on the uterus. It appears that, while JYP had comparable efficacy in improving estrogen deprivation-induced psychiatric symptoms, JYP may possess a better safety profile than estrogen therapy. Such therapeutic advantages also have been observed in several herbal medicines that contain JYP individual materials in OVX+CUMS mice (16, 17) and patients with menopause syndrome and hyperprolactinemia (35, 36).

Ovarian hormones regulate the response of the HPA axis to menstrual cycle-associated stress *via* brain GABAergic neuronal system (19). In this study, we found that ovarian hormone deprivation evoked strikingly elevated serum levels of FSH and LH, which are indicative of ovarian failure, but exhausted blood estradiol level and hypothalamic GABA contents. OVX mice with stress exposure also exhibited markedly elevated serum levels of corticosterone (CORT) and ACTH, the two key stress hormones that play the crucial roles in the pathogenesis of menopausal anxiety and mood symptoms (19, 37). These results support the notion that estrogen deprivation caused anxiety and mood disorders may be derived from fluctuations in female sex hormones that result in altered GABAergic regulation of the HPA axis (19, 37, 38). Chronic E_2_ and JYP in particular the high dose almost completely reversed OVX+CUMS-induced elevated FSH, LH, CORT and ACTH to control levels, and restored the hypothalamic GABA contents. However, unlike E2, JYP did not affect blood estradiol level. JYP appears to mainly modulate the upstream factors of the HPO axis and inhibit the HPA-axis hyperactivity by reinstating the hypothalamic GABAergic neuronal function.

GABA and glutamate are two key neurotransmitters that work together to maintain a balance between excitatory and inhibitory transmission in the brain (39). Dramatic fluctuations in the levels of ovarian hormones during menopause transition profoundly disturbs this functional balance, causing pathological anxiety, mood, and cognitive disorders (40). This study revealed that, in addition to the hypothalamic GABA, ovarian hormone deprivation plus stress exposure also exhausted GABA contents in the hippocampus and prefrontal cortex, but largely restored the glutamate contents and expression of its receptor NMDR1 in the two brain regions, probably causing an imbalance of opposite effects of the two amino acid transmitters. Both E_2_ and the high dose JYP entirely suppressed OVX+CUMS-induced elevation of glutamate contents and NMDR1 expression; however, unlike E_2_, JYP had no significant effects on GABA contents in the two brain regions examined. It seems that JYP may dominantly modulate glutamatergic neuronal functions in the brain regions associated with learning and memory.

Neurotrophins play the crucial roles in the survival, maintenance, and regeneration of specific neurons as well as uterine growth and proliferation (41–43). NGF, GDNF, and BDNF are the three most abundant neurotrophins which widely exist in the adult brain and are highly expressed in the female reproductive system (44, 45). High level of peripheral NGF and BDNF has been proven to be associated with ovarian and breast cancer, polycystic ovarian syndrome (PCOS), and endometriosis (27, 46, 47). Similar to our previous studies (16, 17), this study confirmed that OVX+CUMS profoundly suppressed the expression of the three neurotrophins in the uterus, hippocampus, and prefrontal cortex, but not NGF in the two brain tissues, suggesting that ovarian hormone deprivation may evoke differential effects on brain neurotrophic systems. While the OVX+CUMS-induced decreases of the three neurotrophins in the two brain regions examined were entirely reversed by E_2_ and either or both doses of JYP, E_2_ additionally overturned and even enhanced the expression of the three neurotrophins in the uterus, but JYP did not. It therefore seems that increased risk of breast and endometrial cancer often occurred in estrogen therapy may be related to its enhancement effects on peripheral neurotrophins (48, 49), and the potential better safety profile of JYP observed in this study is, at least in part, derived from it tissue-specific effects on neurotrophins, particularly without effects on uterine neurotrophins.

The estrogen receptors, ERα and ERβ, are closely associated with the pathophysiology of menopause-related metabolic, neurological, and psychiatric disorders (50–52). The two subtypes have distinct anatomical distribution patterns, different physiological processes in the brain and peripheral organs, and even counteract each other (53, 54). There have been contradictory studies on the effects of OVX on the expression of estrogen receptors in different brain regions (54–56). In this study, we revealed that the removal of the ovaries caused a widespread suppression of the expression of the two receptor subtypes across the brain regions examined and the uterus. Chronic E_2_ reversed the OVX+CUMS-induced suppression of the two subtype expression in the uterus, ERα in all the three brain regions, and ERβ in the hypothalamus, but failed to reverse hippocampal and prefrontal ERβ expression. The high dose JYP reversed the OVX+CUMS-induced decreases of hypothalamic and hippocampal ERα. Either or both doses of JYP also prevented ERβ expression in the three brain regions from a combination of OVX and CUMS. However, either dose of JYP had no significant effects on OVX+CUMS-induced changes in uterine ERα and ERβ. These results demonstrated that, like the tissue-specific effects of JYP on neurotrophins, the effects of JYP on the estrogen receptors also seem to be tissue-specific, i.e., JYP may have brain-predominant effects in modulating estrogen receptors, with minor effects or even without effects on estrogen receptors of female peripheral reproductive organs. The aberrant expression of estrogen receptors has been suggested to be associated with ovarian cancer, breast cancer, and other human cancers (57–59). Therefore, the minor and even no effects of JYP on uterine estrogen receptors may be an additional factor contributing to the potential better safety profile of JYP observed in this study.

## Limitations

Several limitations of this study should be considered. First, JYP as a whole preparation was evaluated in this study. We were unable to determine which individual materials or constituents play the principal roles in the psychotropic effects of JYP observed in this study. Further characterization of bioactive constituents of JYP could help better understand a phytochemical profile of this herbal agent, probably resulting in the discovery of novel constituents for treating estrogen deprivation-association disorders in particular psychiatric symptoms. Second, this study did not directly examine the beneficial effects of JYP in improving other estrogen deprivation-related symptoms, such as hot flush and night sweats. Previous studies have revealed the benefits of several herbal preparations in reducing hot flush in menopausal women (5). Finally, we did not consider the effects of the ovarian cycle in the sham mice as control group. Nevertheless, one recent study has shown that there were only differences in the severity of anxiety- and depression-like behavior in female C57BL mice across the estrous cycle (60). It means that we took “average” levels of anxiety and depression across the estrous cycle to serve as control.

Collectively, JYP has comparable efficacy in reducing psychiatric disorders observed in OVX mice exposed to stress with a better safe profile. The therapeutic advantages of JYP may be associated with certain uterus-brain mechanisms distinct from estrogen therapy. It deserves a clinical assessment as an alternative therapy in menopausal women with apparent psychiatric symptoms.

## Data Availability Statement

The raw data supporting the conclusions of this article will be made available by the authors, without undue reservation.

## Ethics Statement

The animal study was reviewed and approved by the Committee on the Use of Live Animals in Teaching and Research of the University of Hong Kong (CULATR 3812-15).

## Author Contributions

X-DZ and Z-JZ were involved in the conception and design of the study, data analysis, and preparation of the manuscript. X-DZ, X-JY, YZ, and Z-SQ developed and conducted experiments. WS provided consultants and technical support. GC provided critical consultants on experiments and critical comments on the manuscript. All authors contributed to the article and approved the submitted version.

## Conflict of Interest

The authors declare that the research was conducted in the absence of any commercial or financial relationships that could be construed as a potential conflict of interest.
